# Metavirome Analysis Reveals a High Prevalence of Porcine Hemagglutination Encephalomyelitis Virus in Clinically Healthy Pigs in China

**DOI:** 10.3390/pathogens12040510

**Published:** 2023-03-24

**Authors:** Weiyao Sun, Zhibin Shi, Pengfei Wang, Bingbing Zhao, Jiaqi Li, Xinyu Wei, Lili Wei, Jingfei Wang

**Affiliations:** State Key Laboratory for Animal Disease Control and Prevention & National Data Center for Animal Infectious Diseases, Harbin Veterinary Research Institute, Chinese Academy of Agricultural Sciences, Harbin 150069, China

**Keywords:** metavirome analysis, swine coronaviruses, nasal swab, serum, PHEV

## Abstract

Six swine coronaviruses (SCoVs), which include porcine epidemic diarrhea virus (PEDV), transmissible gastroenteritis virus (TGEV), porcine hemagglutination encephalomyelitis virus (PHEV), porcine respiratory coronavirus (PRCV), swine acute diarrhea syndrome coronavirus (SADS-CoV), and porcine delta coronavirus (PDCoV), have been reported as infecting and causing serious diseases in pigs. To investigate the genetic diversity and spatial distribution of SCoVs in clinically healthy pigs in China, we collected 6400 nasal swabs and 1245 serum samples from clinically healthy pigs at slaughterhouses in 13 provinces in 2017 and pooled them into 17 libraries by type and region for next-generation sequencing (NGS) and metavirome analyses. In total, we identified five species of SCoVs, including PEDV, PDCoV, PHEV, PRCV, and TGEV. Strikingly, PHEV was detected from all the samples in high abundance and its genome sequences accounted for 75.28% of all coronaviruses, while those belonging to TGEV (including PRCV), PEDV, and PDCoV were 20.4%, 2.66%, and 2.37%, respectively. The phylogenetic analysis showed that two lineages of PHEV have been circulating in pig populations in China. We also recognized two PRCVs which lack 672 nucleotides at the N-terminus of the S gene compared with that of TGEV. Together, we disclose preliminarily the genetic diversities of SCoVs in clinically healthy pigs in China and provide new insights into two SCoVs, PHEV and PRCV, that have been somewhat overlooked in previous studies in China.

## 1. Introduction

Coronaviruses (CoVs) are giant positive-stranded RNA viruses belonging to the family *Coronaviridae*. Coronaviruses’ high mutation rates and recombination propensity enable them to overcome host species barriers and adapt to new hosts [1,2]. The CoVs that have caused three major human epidemics, including severe acute respiratory syndrome virus (SARS-CoV) in 2003 [3], Middle Eastern respiratory virus (MERS-CoV) in 2012 [4], and severe acute respiratory syndrome coronavirus 2 (SARS-CoV-2) in 2019 [5], are believed to have originated from animals. Six species of CoVs are capable of infecting pigs. Four of these, namely transmissible gastroenteritis virus (TGEV), porcine epidemic diarrhea virus (PEDV), porcine respiratory coronavirus (PRCV), and swine acute diarrhea syndrome coronavirus (SADS-CoV), belong to the genus *Alphacoronavirus*; the other two, porcine hemagglutination encephalomyelitis virus (PHEV) and porcine delta coronavirus (PDCoV), are members of the genus *Betacoronavirus* and *Deltacoronavirus*, respectively [6].

The SCoVs, including PEDV, PDCoV, SADS-CoV, and TGEV, are the main porcine enteric coronaviruses, causing severe diarrhea in pigs with high mortality rates and responding to significant economic losses in the swine industry [6]. After being infected with those viruses, pigs exhibit similar clinical symptoms, mainly including anorexia, diarrhea, vomiting, dehydration, weight loss, lethargy, and death [7]. Clinical symptoms typically appear 1–3 days after infection and usually do not last longer than 10 days. The disease is usually fatal in neonatal pigs, especially those born from seronegative mothers. In older pigs, the incidence of the disease is higher, but the mortality rate is lower [8]. The viruses infecting pigs did not display high viral loads at their replication sites, but these could be detected in other extra-replication sites, such as respiratory tissues [9,10,11]. PEDV first appeared in the United Kingdom and Belgium in the early 1970s [12,13] and has sporadically circulated in China and other countries for over 40 years [14]. Since the outbreak of new PEDV strains in southern China after 2010, they rapidly spread to other regions and were the most frequently detected viruses in diarrheal pigs, with a prevalence of up to 50% in some areas in southern China from 2012 to 2018 [15,16,17]. This resulted in large-scale outbreaks with incidence rates of 80–100% among infected pig herds and a mortality rate of 50%–90% among suckling piglets [18,19,20]. The new emerging PDCoV, first identified in Hong Kong in 2012 [21], was listed as the second most common pathogen causing swine diarrhea [22,23]. To date, PDCoV has been reported in the US, Canada, Korea, China, and Thailand [23,24,25], with a detection rate of about 30% in swine diarrhea samples in China in 2015 [26]. SADS-CoV was first detected in Guangdong province of China in 2017, causing the death of approximately 24,500 piglets, and reemerged in pig herds in this province in February 2019, resulting in the death of about 2000 pigs [27,28]. SADS-CoV has not been reported in other provinces in China or in any other regions of the world. TGEV was found to have a lower incidence of less than 3% in China in recent years, while in the United States, the prevalence of TGEV decreased from 6.8% to 0.1% from 2010 to 2016 [29,30]. From an epidemiological perspective, it is of extreme importance to note the existence of cross-resistance between PRCV and TGEV, which could be the reason for the elimination of TGE and is no longer a significant issue for pig farmers in Europe [31].

PRCV and PHEV are non-enteropathogenic SCoVs. PRCV is a naturally occurring spike gene deletion mutant of TGEV and preferentially infects the respiratory system, causing dyspnea, sneezing, and tachypnea in infected pigs with a negligible mortality rate [32,33,34]. It spread quickly in European pig populations without causing significant health problems [6]. PHEV is one of the first swine coronaviruses to be discovered and isolated, and the only neurotropic virus known to affect porcine populations [32]. It is able to infect naive pigs at any age, but clinical symptoms caused by PHEV are variable [35]. PHEV infection causes vomiting and wasting with a high mortality rate in piglets, primarily those under three weeks. In contrast, PHEV infection presents subclinical manifestations in growing and adult pigs [36]. Moreover, PHEV is highly prevalent and circulates in most pig-producing regions of the world, including Europe, the Americas, Asia, and Australia [32,37].

Next-generation sequencing (NGS) technology has developed rapidly over the past 15 years. The advantages of the high throughput and low cost of this technology have promoted the development of genomics in clinical medicine, biomedicine, and other research fields. In terms of animal pathogen research, its usage enables the simultaneous detection of the gene of multiple microorganisms and the identification of not only known pathogens but also novel or unidentified potential pathogens [38,39,40]. Combining this technology, many swine viruses, such as PEDV [41] and ASFV [42,43], have been identified in several previous studies using metavirome analysis, which will help us to view the overall perspective of the composition of the viral community that infects pigs and other animals in the future studies.

In this study, we collected nasal swabs and serum samples from clinically healthy pigs in China in 2017 and performed metavirome analysis for SCoVs. We found five species of SCoVs, of which PHEV was the most abundant, circulating in healthy pig farms and then performed phylogenetic analysis for each detected species of SCoVs. The discovery and phylogenetic analysis of these coronaviruses provides new insights into the comprehensive understanding of the porcine coronavirus community and provide early warning for outbreaks of porcine coronavirus diseases.

## 2. Materials and Methods

### 2.1. Ethics Statements

This disease surveillance program and sampling strategy were approved by the Animal Husbandry and Veterinary Bureau of the Ministry of Agriculture and Rural Affairs of the People’s Republic of China. 

### 2.2. Sample Collection

**Figure 1:**
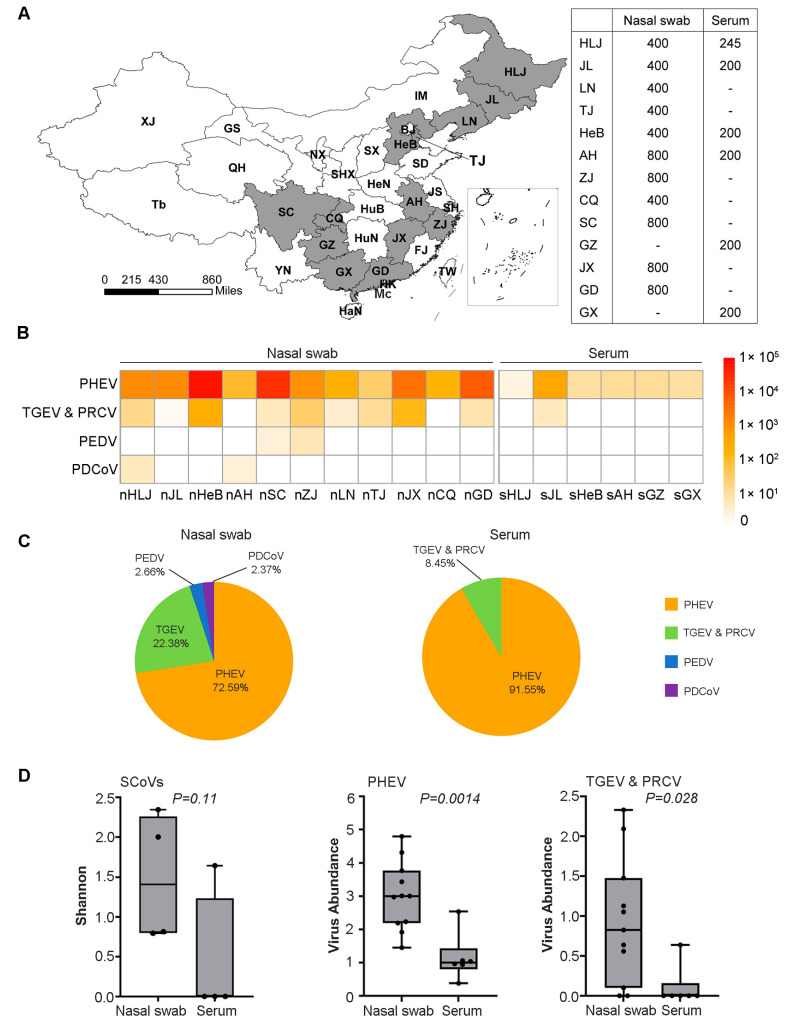
Geographical distribution of the samples and abundance analysis of the SCoVs. (**A**) Map of sampling province and sample size. Provinces with samples collected are colored dark gray. “-” represents no sample collected. Abbreviations: HLJ (Heilongjiang); JL (Jilin); LN (Liaoning); BJ (Beijing); TJ (Tianjin); IM (Inner Mongolia); HeB (Hebei); SD (Shandong); SX (Shanxi); HeN (Henan); JS (Jiangsu); AH (Anhui); SH (Shanghai); ZJ (Zhejiang); FJ (Fujian); JX (Jiangxi); GD (Guangdong); GX (Guangxi); HuB (Hubei); HuN (Hunan); GZ (Guizhou); SC (Sichuan); CQ (Chongqing); SHX (Shaanxi); NX (Ningxia); GS (Gansu); QH (Qinghai); XJ (Xinjiang); Tb (Tibet); YN (Yunnan); HaN (Hainan); TW (Taiwan); HK (Hong Kong); Mc (Macao). The map was generated using QGIS3. (**B**) The heatmap of the SCoVs gene abundance from 17 libraries. The abundance levels range from 0 to 1 × 10^5^ RPM. n (nasal swab); s (serum). (**C**) Pie chart shows the proportion of the gene abundance of PEDV, TGEV, PEDV, and PDCoV. (**D**) Alpha-diversity and viral abundance between the two sample types and the Shannon diversity index represents the diversity level, and the virus abundance unit is log_10_ (RPM). Each box shows the estimates median, upper and lower quartiles, and the black circle represents each library. The *p* value is labeled above the box.

A total of 6400 nasal swabs and 1245 serum samples were collected from clinically healthy pigs at slaughterhouses in 13 provinces in 2017 (Figure 1A). Each of the nasal swabs was immersed in 1.5 mL sterile phosphate-buffered saline (PBS), and each of the serum was stored in 1.5 mL microcentrifuge tube. These samples were kept at −20 °C until testing.

### 2.3. Sample Preparation and Sequencing

The samples were pooled by type and province into 17 pools. Sample processing and viral nucleic acid library preparation were performed as previously described [44,45]. Briefly, the swab solutions and serum were centrifuged at 13,000× *g* for 20 min to remove impurities, and the supernatants were then filtered through a 0.45-μm- and a 0.22-μm-filter sequentially. The pellet was immersed in PBS overnight after centrifuging at 160,000× *g*, 4 °C for 4 h. The precipitates were repeatedly admixed and dissolved in PBS. To eliminate the contamination of exogenous nucleic acid, the samples were treated with DNase I (70U/μL, Takara, Dalian, China) and RNase I (10U/μL, Thermo scientific, Waltham, MA, USA) at 37 °C for 2 h. Viral DNA/RNA was extracted from samples using an EasyPure Viral DNA/RNA kit (TransGen, Beijing, China). A random PCR process was then performed as follows: the first strand cDNA was synthesized with the random primer of K9N (5′-GACCATCTAGCGACCTCCCANNNNNNNNN-3′) and PrimeScript II RTase (Takara, Dalian, China) at 42 °C for 3 h and then inactivated at 95 °C for 5 min; the second strand cDNA was synthesized with DNA Polymerase I Large (Klenow) Fragment (Promega, Madison, Wisconsin, USA) at 37 °C for 1 h, and then inactivated at 75 °C for 10 min. The DNA/cDNA was then amplified in a total reaction volume of 50 μL, including 2 × KOD FX Neo buffer, 0.5 mmol/L each dNTP, 5 μL nucleotide, 10 mmol/L random primers of K9 (5′-GACCATCTAGCGACCTCCCA), and 1U KOD FX Neo DNA polymerase (Toyobo, Osaka, Japan). Finally, amplification was performed with 1 cycle of 94 °C for 2 min, followed by 40 cycles of 98 °C for 10 s, 55 °C for 30 s, and 68 °C for 2 min. The PCR products were assessed by electrophoresis (1% agarose gel). A total weight of 6 μg of random PCR products of 17 samples were submitted to Shanghai Personalbio Company and sequenced using the Illumina HiSeq 2500 platform (PE 2 × 150 bp).

### 2.4. Viral Genome Discovery

For each library, the quality control of sequencing reads was performed with the BBTools software package (http://www.jgi.doe.gov/data-and-tools/bbtools/ (accessed on 10 March 2020)) and rRNA associated reads were removed by mapping reads against a comprehensive rRNA collection downloaded from the SILVA database (https://www.arb-silva.de/ (accessed on 15 March 2020)) using bowtie2 software (v1.2.2) [46]. The remaining reads were compared against the local constructed database based on the virus sequences belonged to the family *Coronaviridae* with an evaluated threshold of 1 × 10^−4^. The abundance for each viral taxonomy was estimated as reads per million (RPM), and only those with RPM > 1 were considered positive. To obtain longer virus sequences for phylogenetic analysis, rRNA-free reads that were successfully matched to the reference viral genomes were assembled de novo using the megahit program (v1.2.6) [47]. The parameters were set for blastn and diamond blastx taxonomy annotations, as described in the previous section. Viral contigs were confirmed to the reference viral sequences with bowtie2 again.

### 2.5. Phylogenetic Analysis

All the sequences were aligned by MAFFT algorithm in Geneious Prime (v 2022) [48], and the phylogenetic trees were then constructed using the maximum likelihood approach implemented in MEGA (v 6.06) with a bootstrap value of 1000, which ensures the reliability of the branching orders [49]. SimPlot (v3.5.1) was used to generate a similarity map of full-length PHEV genome [50].

### 2.6. Nucleotide Sequence Accession Number

The complete genome sequences of PHEV/GD/2017, PHEV/HLJ/2017, PHEV/SC/2017, and PHEV/ZJ/2017 have been submitted to the GenBank database with accession number OQ305205, OQ305206, OQ305207, and OQ305208, respectively.

## 3. Results

### 3.1. Diversity and Phylogenetic Evolution of SCoVs

To survey the genetic diversity and prevalence of SCoVs in pigs in China, we constructed and sequenced 17 libraries, through eleven nasal swabs and six serum samples, which represented more than 7500 clinically healthy pigs from 13 provinces in China (Figure 1A). Four species of SCoVs, including PHEV, TGEV, PEDV, and PDCoV, were identified by BLAST analysis (Figure 1B). Of these, PHEV was the most abundant species and was detected in all of the nasal swabs and serum samples collected in the thirteen provinces and TGEV was the second most abundant species detected in eight provinces, whereas PEDV and PDCoV had the lowest abundance and were only detected in nasal swabs of two provinces (Figure 1B,C). The alpha diversity of SCoVs measured by the Shannon index showed no significant difference (*p* = 0.11) between these two types of samples, but the viral genome sequence abundance in the nasal swabs was significantly higher than that in the sera for PHEV (*p* = 0.0014) and TGEV (*p* = 0.028) (Figure 1D). These results showed that PHEV and TGEV were more prevalent than PEDV and PDCoV in clinically healthy pigs in China.

**Figure 2:**
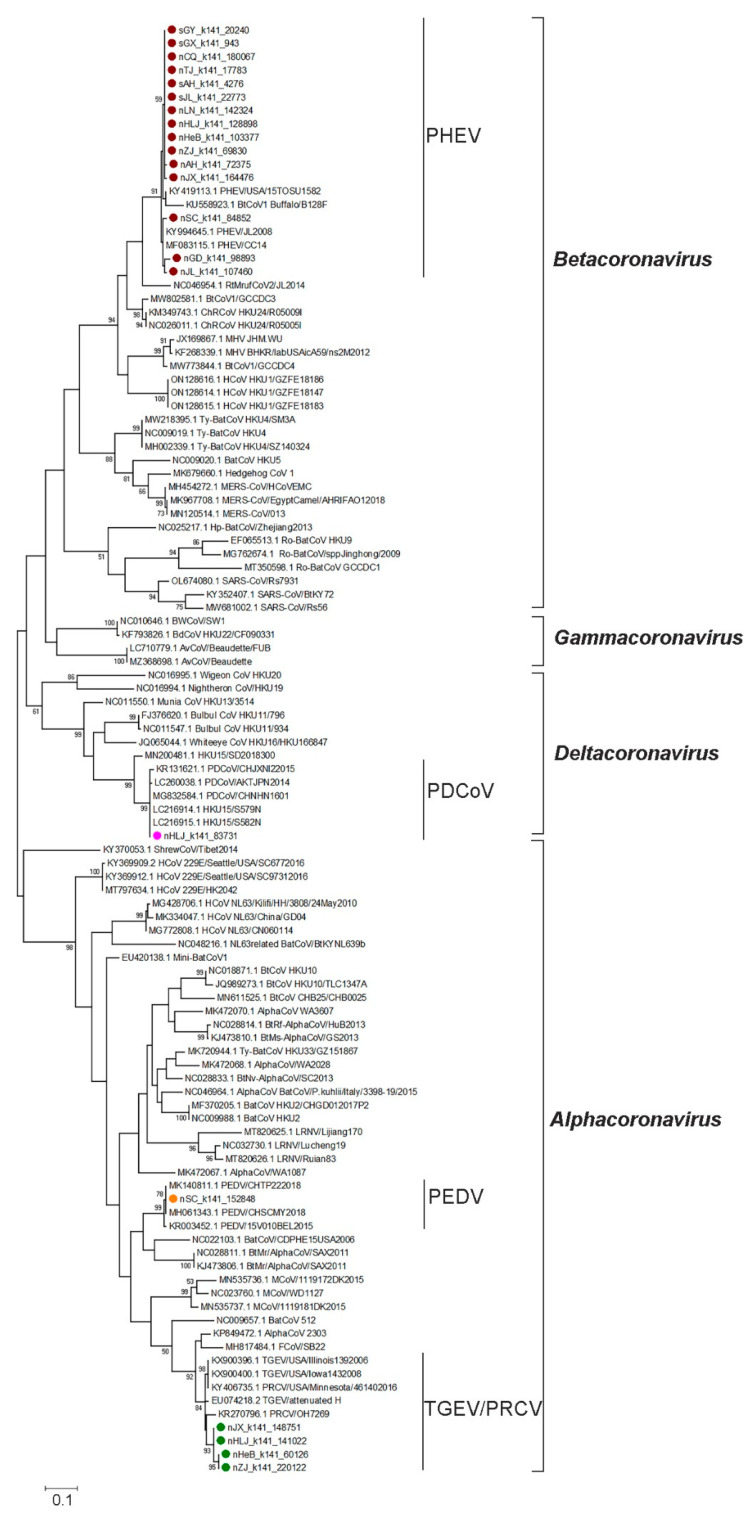
Maximum likelihood tree of SCoVs. The maximum likelihood tree of the viruses in the family *Coronaviridae* was constructed based on the partial ORF1b (18,482~18,775 nt, referenced according to the genome sequence of PHEV isolate CC14) using MEGA 6.06 and tested with 1000 bootstrap replicates. PHEV, TGEV/PRCV, PEDV, and PDCoV identified in this study are labeled with red, green, orange, and pink solid circles, respectively. The names of the genus and species of SCoVs are labeled on the right of the corresponding branches.

To further explore the genetic evolutionary characteristics of SCoVs identified in this study, we constructed a maximum likelihood tree based on the partial ORF1b gene (18,482~18,775 nt) of 19 SCoVs identified in this study and 87 reference strains (including 42 alphacoronaviruses, *29* betacoronaviruses, four gammacoronaviruses, and 12 deltacoronaviruses) in the family of *Coronaviridae*. In the ML tree, different species of the reference SCoVs were successfully clustered into different branches, which helped us to identify the species of the SCoVs obtained in this study (Figure 2). Notably, 13 strains of the new identified SCoVs were divided into the *Betacoronavirus* branch and showed close relationships with the previously identified PHEVs. Four new SCoVs were assigned to the TGEV and PRCV subgroup in the *Alphacoronavirus* branch. While the remaining two strains showed high homology with the PEDV in the *Alphacoronavirus* clade and PDCoV in the *Deltacoronavirus* clade, respectively. The phylogenetic analysis results based on this gene fragment supported the conclusion that we were able to identify at least four species of SCoV in this study.

### 3.2. Genetic Diversity and Evolution of PHEV

**Figure 3:**
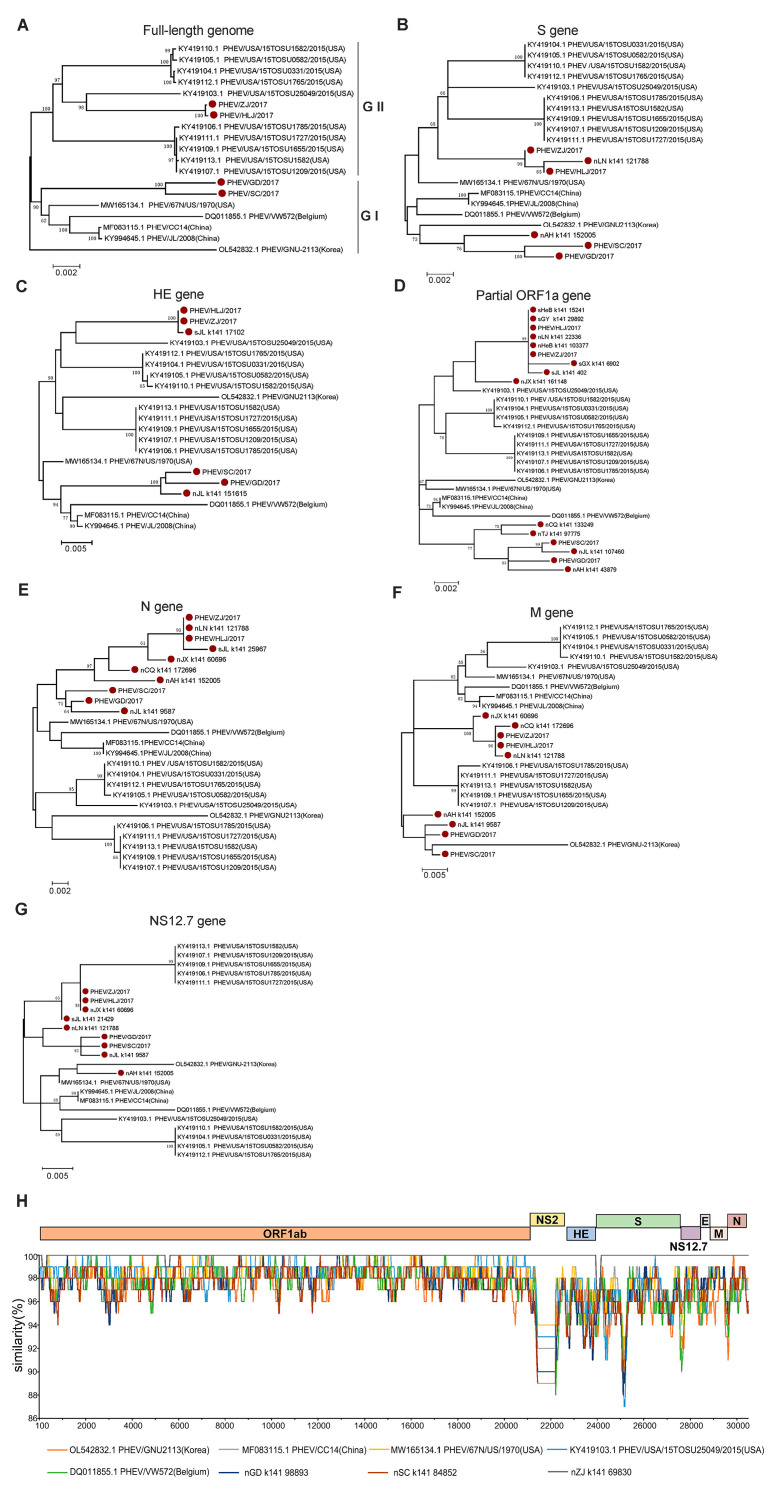
Evolutionary analysis of PHEV. The maximum likelihood trees based on the full-length genome sequences (**A**), the S gene sequences (**B**), the HE gene sequences (**C**), partial ORF1a gene fragments (**D**), the N gene sequences (**E**), the M gene sequences (**F**), and NS12.7 gene sequences (**G**) of PHEV were built using MEGA 6.06 with 1000 bootstrap replicates. PHEV identified in this study were labeled with red solid circles. (**H**) Similarity analysis of the full-length genome of representative PHEVs. The genome sequence of PHEV/HLJ/2017 was used as the query to compare with the other eight representative strains.

PHEV was proven to be the most widespread species of SCoV in the clinically healthy pigs in this study. To understand the evolution of the PHEVs, an ML tree was constructed based on the full-length genome sequences of four PHEVs obtained in this study (namely PHEV/HLJ/2017, PHEV/ZJ/2017, PHEV/SC/2017, and PHEV/GD/2017) and fifteen reference strains were downloaded from the GenBank database. The result showed that these PHEVs were classified into two major branches, GI and GII. The GI group included the viruses identified from Belgium (two), the US (one), and China (four, including two identified in this study). The GII group was dominated by the viruses from the US (ten) and also included two new Chinese PHEVs. A strain from Korea (GNU-2113) was not located in either of the two groups, indicating that this virus evolved independently (Figure 3A).

Given very little knowledge is available concerning PHEVs in China, we further explored in detail the evolution of PHEVs through phylogenetic analyses based on different gene segments of the PHEVs. Phylogenetic trees based on the genes S (Figure 3B), HE (Figure 3C), and partial ORF1a (Figure 3D) showed a similar topology to those based on the full-length genome sequences, in which the PHEVs were clustered in two major branches: one branch consists mainly of the US isolates and the new viruses identified in this study; while another branch is composed mainly of viruses previously found in China and those originating in countries such as Belgium. However, in the ML tree based on the N gene sequences, all the newly identified viruses were clustered in one branch and showed a close relationship with viruses isolated from the US, Belgium, and China (Figure 3E). The ML tree based on the M gene sequences assigned the viruses into four branches and the Chinese viruses were clustered in two independent branches. Interestingly, the PHEVs from the US that clustered in one branch in other trees were also divided into two evolutionary branches (Figure 3F). The PHEVs were also divided into two major groups according to the ML tree based on the NS12.7 gene sequences, but the virus composition in each branch is largely different from that of the phylogenetic tree based on the full-length genome sequences, and the majority of PHEVs identified in this study showed a closer evolutionary relationship with some viruses from the US (Figure 3G). Together, these results indicate a complex evolutionary trace and high genetic diversity of PHEVs in China.

To further verify the phylogenetic analysis results, we conducted a recombinant analysis based on the four PHEVs identified in this study and five reference strains. The result showed that the ORF1ab encodes the non-structural proteins and was relatively conserved among all the viruses. However, all the genes that encode structural proteins and genes encoding no structural proteins NS2 and NS12.7 showed a higher variation; however, recombination has not been detected among these viruses (Figure 3H).

### 3.3. Genetic Diversity of Other SCoVs

**Figure 4:**
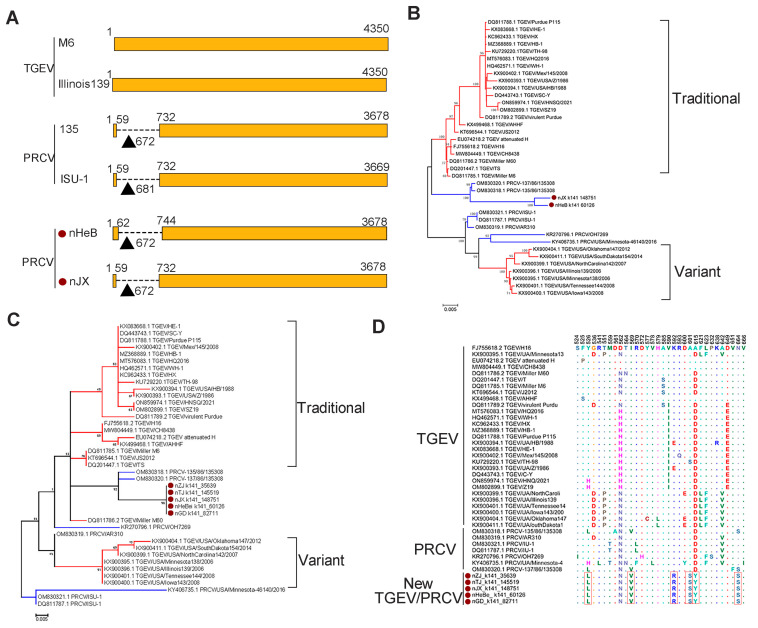
Evolutionary analyses of TGEV and PRCV. (**A**) Schematic diagram of the S gene of TGEV and PRCV. The deletion region in the S gene is shown as a dotted line. The TGEV strain Miller M6 was used as the reference strain. The PRCVs identified in this study are labeled with red circles. Phylogenetic trees of TGEV and PRCV based on the S gene (**B**), and the amino acid sequences of RBD (**C**) were constructed using the maximum likelihood method with 1000 bootstrap replicates implemented in MEGA 6.06. The branches colored red and blue represent TGEVs and PRCVs, respectively. (**D**) Amino acid mutations in the RBD sequences of the TGEV and PRCV. New viruses identified in this study are marked with red circles and distinct amino acids in the RBD sequences of the new viruses are indicted with red boxes.

The PRCV is a naturally occurring spike gene deletion mutant of TGEV. To confirm the presence of PRCV among the viruses that were classified as TGEV by BLAST analysis, we conducted the multiple sequence alignment of all the S genes obtained in this study and found the deletion existed in two sequences (Figure 4A), suggesting that we identified at least two PRCVs among the TGEVs. To explore the phylogeny of the PRCVs, we built an ML tree based on the S gene sequences of these two viruses and 36 reference strains, which showed that the two PRCVs were clustered with two previously identified Chinese strains together in one sub-branch. The newly identified PRCVs were phylogenetically closer to traditional TGEVs, rather than its variants (Figure 4B). The phylogenetic tree based on the amino acid sequences of receptor-binding domain (RBD) of the S protein also showed a similar phylogeny with that based on the S gene sequences: all viruses identified in this study, together with two previous PRCV stains (OM830318.1 and OM830320.1), formed one subbranch and showed a close relationship with traditional TGEVs (Figure 4C). To further explore the potential differences among these viruses with receptor-binding properties, we analyzed the amino acid variation in the RBD of the S protein of the viruses identified in this study and 37 reference viruses. The result showed that six distinct amino acid substitutions, including Y526L, I569V, K592R, A601S, D615Y, and N664S, occurred in the five newly identified viruses, suggesting that these viruses may have different receptor-binding properties (Figure 4D).

**Figure 5:**
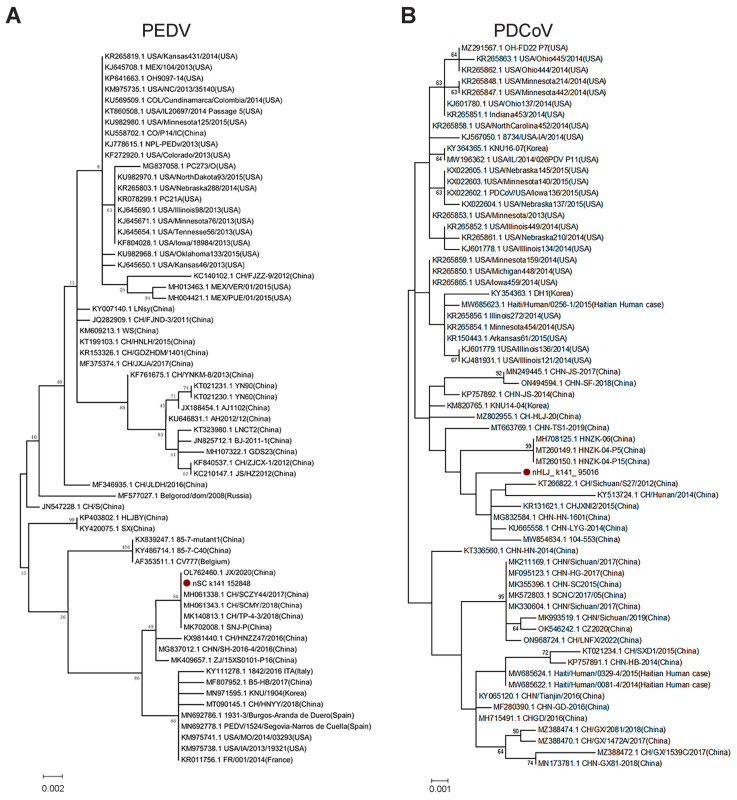
Phylogenetic trees of PEDV and PDCoV. (**A**) The partial ORF1b gene (sequence 17,746~18,510 nt, referenced according to the genome sequence of PEDV isolate CV777) tree of PEDV. (**B**) PDCoV tree based on nucleotide segments (sequence 23,645~24,742 nt, referenced according to the genome sequence of PDCoV isolate CHN-HN-1601). PEDV and PDCoV identified in this study were labeled with red solid circles. The trees were constructed using the maximum likelihood method with 1000 bootstrap replicates implemented in MEGA 6.06.

Although PEDV and PDCoV are two of the most serious disease-causing SCoVs in pigs, they had the lowest abundance in this study. The number of contigs belonging to the genome of PEDV and PDCoV was only 10 for each, respectively, and these contigs are located in the different regions of the viral genome. Therefore, they could not be used to conduct phylogenetic analysis systematically, and we selected one contig, respectively, for the construction of the PEDV or PDCoV ML tree. Preliminarily, these trees demonstrated that the new PEDV (Figure 5A) and PDCoV (Figure 5B) had a close evolutionary relationship with corresponding Chinese isolates.

## 4. Discussion

SCoVs, such as PEDV, PDCoV, and TGEV, are pathogens causing significant economic losses in the global swine industry. The genetic diversity of SCoVs has not been systematically investigated in China. Several studies have reported the viral diversity of SCoVs in fecal tissues collected from pigs with symptoms of diarrhea [45,51]. In this study, we conducted a metavirome analysis on the SCoVs in clinically healthy pigs from 13 major pig-producing provinces in China, and five species SCoVs, including PHEV, TGEV, PRCV, PEDV, and PDCoV, were found to be circulating in the pig populations in China, with differences in prevalence and spatial distribution. Most importantly, we identified PHEV, a previously ignored SCoV, in all samples collected from 13 provinces in very high abundance. These findings provide an overview of the circulation status of SCoVs in the pig population in China and useful information for making SCoV prevention and control measures as well.

The epidemiological investigation of the prevalence of severe disease-causing SCoVs has been extensively conducted in many pig production countries. According to these investigations, SCoVs, including PEDV, PDCoV, and TGEV, have been the dominant viral agents for causing diarrhea in pigs, especially newborn piglets [6,12,13,23]. In China, surveys that have reported SCoV infections in pigs were mostly aimed at investigating the cause of diarrhea and revealed a high prevalence in affected pig populations [17]. These studies have addressed the importance of disease-causing SCoVs from a disease-control perspective but ignored the potential influence of other pig-carried coronaviruses. Given the current global public health catastrophe caused by coronaviruses, it is important to know the potential impact of CoVs carried by other animals on the health of both livestock and humans. The overview of infection and genetic diversity of SCoVs in pigs in China, especially in clinically healthy pigs with potentially close contact with humans, was largely unknown. In the current study, we provide for the first time an overview of SCoV infections in clinically healthy pigs in China and identified five species of SCoVs circulating in the pig populations. In particular, PHEVs were found to be widespread and highly prevalent in pig populations. Therefore, the potential impact of these SCoVs on pig production and public health needs to be further evaluated in the future.

Unlike those severe disease-causing SCoVs, such as PEDV, PDCoV, and TGEV [52,53,54,55], PHEV has received less attention due to its low pathogenicity and the fact that it causes only mild clinical changes. To date, most of the PHEV reports have been released from the US, and the prevalence of the virus was over 50% in pig populations of the country [56]. In China, very limited information on PHEV is available and these data showed that PHEV had spread in Changchun City and Siping City of Jilin province with mortality rates of 47.6 and 100%, respectively [57,58]. In this study, we identified PHEV from all the samples collected in 13 provinces, suggesting it was widespread in the pig population in China. Furthermore, we proved that PHEV can be excreted from both the respiratory tract and blood and that there is no difference between these two routes when evaluated in terms of viral abundance, suggesting that both nasal swabs and sera can be used to survey the PHEV infection in pigs. We also obtained four full-length genomes of PHEV and proved for the first time that at least two lineages of the viruses were cocirculating in the pig population in China. Phylogenetic analyses based on the gene sequences, especially those encoding the structural proteins, revealed more complex evolutionary patterns of the viruses, indicating that PHEVs have been undergoing continuous evolution during the circulation [59]. Several studies have reported that interspecies jumping or intraspecies recombination occurred in coronaviruses [60,61], such as PEDV [62] and bat coronaviruses [63]. The high prevalence and variation of PHEVs emphasize that adequate attention should be paid to their potential for cross-host transmission and an increase in pathogenicity in pigs.

Disease-causing SCoVs, such as TGEV, PEDV, and PDCoV, are widespread pathogens in Europe [64], Asia [65], and North America [66]. In our investigation, we found that TGEV had a medium abundance when compared to the other SCoVs identified in this study and was detected in nine provinces, suggesting that it was highly prevalent in clinically healthy pig populations. As for PEDV and PDCoV, our data revealed a low viral load in the two samples, respectively. These results seem different from those reported in previous studies in China, which showed a very high prevalence for PEDV (~55%) and a very low prevalence (~1.0%) for TGEV and PDCoV in pigs with diarrhea [67]. These differences may have resulted from the differences in sampling strategies, sample processing, and detection methods. In this study, we collected samples from clinically healthy pigs, and this was probably the one of reasons for the low abundance of those disease-causing SCoVs detected. Metavirome analysis has advantages over traditional methods, such as RT-PCR, when detecting CoVs, as it detects all viruses in an unbiased manner, while traditional methods are designed to detect specific gene targets.

PRCV is derived from TGEV with a spike protein deletion mutant [68]; therefore, it can be distinguished from TGEV by comparing the S genes. In this study, we detected a 672nt deletion at the N-terminal end of two S gene sequences, proving they were from PRCV. The interaction between the S protein and host cell receptor aminopeptide N (pAPN) facilitates the coronaviruses to enter their host cells [69,70]. Our study found that six amino acid mutations occurred in the receptor-binding domain (RBD) of the S gene of TGEVs or PRCVs, and their impact on receptor-binding properties should be explored in the future.

Overall, in order to investigate the prevalence and genetic diversity of SCoVs in clinically healthy pigs in China, we collected nasal swabs and serum samples from pigs in slaughterhouses throughout China and assessed the potential disease risks for pigs and humans posed by the viruses, especially coronaviruses, carried by those clinically healthy pigs. However, although the respiratory system was identified as containing the extra-intestinal sites for the replication of enteric SCoVs, such as TGEV [11] and PEDV [71,72], those two viruses mainly replicate in the gut tissues, so it is difficult to ascertain the prevalence of these viruses using these types of samples, which is why their abundance is relatively low in this research. The limitations of this study are the limited forms of sampling, resulting in the inability to fully reflect the changing trends of SCoVs in the clinically healthy pig population in China. Subsequent studies should increase the types of collection samples in order to make the research more complete.

## 5. Conclusions

We applied the next generation sequencing and metavirome analysis to explore the diversity of swine coronaviruses in clinically healthy pigs in China. Five species of SCoVs were detected in this study, of which the PHEV was the most abundant species and was widespread in all 13 provinces. We further revealed the genetic evolution and characteristics of these viruses. These findings provide a more comprehensive understanding of the SCoVs community in healthy pigs and emphasize the potential threat of PHEVs to animal and human health.

## Data Availability

The complete genome sequences of 4 PHEV have been submitted to the GenBank database (NCBI) with accession number OQ305205, OQ305206, OQ305207, and OQ305208, respectively.
